# Basic Mechanical Properties and Microscopic Characteristics of Alkali-Activated Slag/Fly Ash Concrete Incorporating Dredged Sand

**DOI:** 10.3390/ma19214668

**Published:** 2026-11-02

**Authors:** Wei Wang, Jinming Yin, Jifeng Yuan

**Affiliations:** Taizhou Institute of Science and Technology, Nanjing University of Science and Technology, Taizhou 225300, China; 22011007@nustti.edu.cn (W.W.); 22006005@nustti.edu.cn (J.Y.)

**Keywords:** alkali-activated slag/fly ash concrete, dredged sand, workability, microscopic characteristics

## Abstract

To improve the utilization efficiency of dredged sand and alleviate the depletion of natural aggregate resources, ultrafine dredged sand (DS) was used as a fine aggregate replacement to prepare alkali-activated slag/fly ash concrete. The effects of DS and fly ash (FA) replacement rates on the workability and mechanical properties of the concrete were investigated. XRD, SEM, and MIP tests were conducted to analyze the hydration products, micromorphology, and pore structure, and the underlying modification mechanism of DS for alkali-activated concrete was clarified. The results show that, compared with the group without dredged sand, the 3 d, 28 d, and 90 d cubic compressive strengths of the group with 30% dredged sand increased by 8.35%, 9.3%, and 9.8%, respectively. Appropriate incorporation of dredged sand can optimize the pore distribution and microstructure, improve the compactness of the concrete matrix, and significantly enhance both the early and later strength of alkali-activated concrete. The 28 d compressive strength and splitting tensile strength of AASC both increase first and then decrease with the rising FA dosage. Moreover, the incorporation of fly ash exerts a remarkable enhancement effect on its later strength.

## 1. Introduction

The Yangtze River basin is known as China’s “golden waterway”. To meet navigation requirements, the river is dredged regularly every year. During the dredging process, a large amount of dredged sand is generated, which is often made of small particles and is difficult to utilize [1,2]. The conventional disposal method for dredged sand is dumping in areas designated by waterway authorities. However, adverse impacts on the river environment and riverine ecosystems are exerted by sand dumping, and the effectiveness of waterway dredging is also impaired. Therefore, it is necessary to develop a new method for the treatment of these dredged sands [3,4,5,6].

Concrete is one of the most widely used materials in the construction industry. However, with the gradual depletion of natural sand resources worldwide, high-quality fine aggregates have become difficult to obtain. The shortage of fine aggregates for concrete is particularly severe in developing countries [7,8]. Alkali-activated slag concrete (AASC) adopts ground granulated blast-furnace slag (GGBS) as a substitute for cement to lower the cement dosage and mitigate carbon emissions [9]. The incorporation of Yangtze River dredged sand as a fine aggregate into AASC facilitates the fabrication of a novel eco-friendly concrete, which achieves green production for both binders and aggregates. In recent years, research on the application of dredged sand in concrete has mainly focused on three aspects: property characterization of dredged sand, investigation of mechanical performance, and analysis of microscopic mechanisms.

Material properties of dredged sand are vital parameters for studying material characteristics. Wu Chaoguo [10] selected nine characteristic parameters to characterize the shape and angularity features of dredged sand. Meanwhile, a comprehensive model for the overall evaluation of different fine aggregates was established based on the gray relational analysis method. The research results show that using dredged sand to replace part of river sand as a fine aggregate for concrete preparation can increase the overall coefficient of fine aggregates. Based on a dredging project of a terminal in Jiangsu Province, Zhang Shilei [11] collected sand samples at different depths and conducted particle size analysis and mud content tests to further reveal the particle composition of Yangtze River sand. The experimental results show that the dredged sand in the lower reaches of the Yangtze River is fine-grained, with particle sizes ranging from 0.15 mm to 0.30 mm. The mud content in deep-layer sand is relatively low, and the dosage of ultrafine sand needs to be controlled in engineering applications. Zang Yingping [12] analyzed the pH value, chemical composition, mineral composition and particle size distribution of waste ultra-fine sand from the middle and lower reaches of the Yangtze River. The results show that the waste sand from this waterway is slightly alkaline. Non-clay minerals such as hydromica account for 84%, while clay minerals including vermiculite and chlorite make up 16%. The particles are mainly distributed in the range of 0.15–0.30 mm with a fineness modulus of approximately 0.8, which falls into the category of ultra-fine sand. The soil sample exhibits low reactivity.

Mechanical performance constitutes one of the core research directions of concrete. Zril [13] prepared sandy concrete using waste sandy soil, cement, mineral fillers, and chemical admixtures, and the prepared concrete achieved a compressive strength of 41 MPa. Wu Jing [14] adopted dredged sand from the lower reaches of the Yangtze River to replace fine aggregate in concrete for the fabrication of alkali-activated slag concrete. The dynamic mechanical properties of concrete with five different replacement ratios of dredged sand were investigated. The results demonstrate that alkali-activated slag concrete incorporating dredged sand from the lower Yangtze River is a strain-rate-sensitive material, and the peak stress rises with the increase in strain rate. Guo Wenwen [15] utilized waste ultrafine sand generated in waterway regulation works to produce sandy concrete. The 28-day compressive strength of the resulting concrete exceeds 30 MPa. This material can replace ordinary concrete for manufacturing ballast blocks and be locally applied in general water transport engineering.

Investigations into the microscopic mechanisms of dredged sand concrete have been reported, but the available research is relatively limited. Pan Yuepeng [16] prepared sandy concrete using waste ultrafine sand from waterway regulation projects. X-ray diffraction (XRD) technology was adopted to characterize the hydration products of the fabricated concrete. The results indicate that the sandy concrete contains abundant hydration products, including calcium silicate hydrate (C-S-H) and ettringite. Li Shengtao et al. [17] analyzed the phase composition of AASC through X-ray diffraction (XRD) and scanning electron microscopy (SEM). The results show that the incorporation of dredged sand does not generate new crystalline phases. However, their study failed to consider the influence of fly ash dosage on the performance of alkali-activated concrete. Xu Huiying [18] reconstructed the three-dimensional model of dredged sand through CT technology and investigated the evolution law of the pore structure of dredged sand under freeze–thaw cycles. Furthermore, SEM and XRD tests were conducted to analyze the morphology and phase composition of dredged sand concrete. The research results show that dredged sand affects the performance of concrete mainly through physical action.

This study aims to fully utilize waste ultrafine sand generated during channel dredging to effectively alleviate the shortage or depletion of natural aggregate resources. Nevertheless, the engineering application of ultrafine dredged sand still faces several limitations, particularly regarding the dosage optimization and microscale interaction mechanisms in alkali-activated concrete. In this paper, GGBS and (FA) are adopted as alkali-activated precursors. The effects of ultrafine DS and FA on the mechanical properties and microstructure of alkali-activated concrete are investigated, and the underlying mechanisms by which ultrafine dredged sand and fly ash regulate material performance are further revealed. The findings of this study can provide a scientific basis for the engineering application of ultrafine dredged sand as an alternative fine aggregate in alkali-activated materials. This work facilitates the disposal of waste dredged sand from waterways, prevents environmental pollution, and offers an effective route for the resource utilization of waste ultrafine sand.

## 2. Materials and Methods

### 2.1. Materials

**Figure 1:**
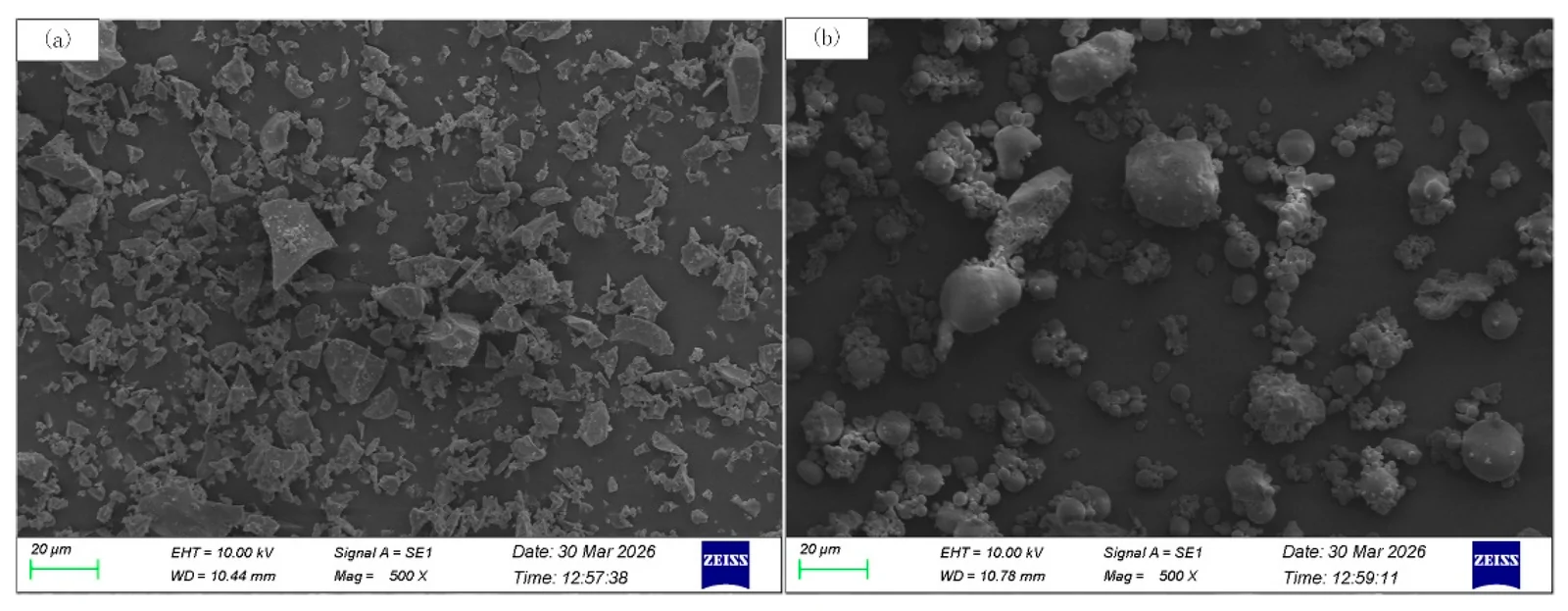
SEM images of raw materials (**a**) GGBS and (**b**) FA.

**Figure 2:**
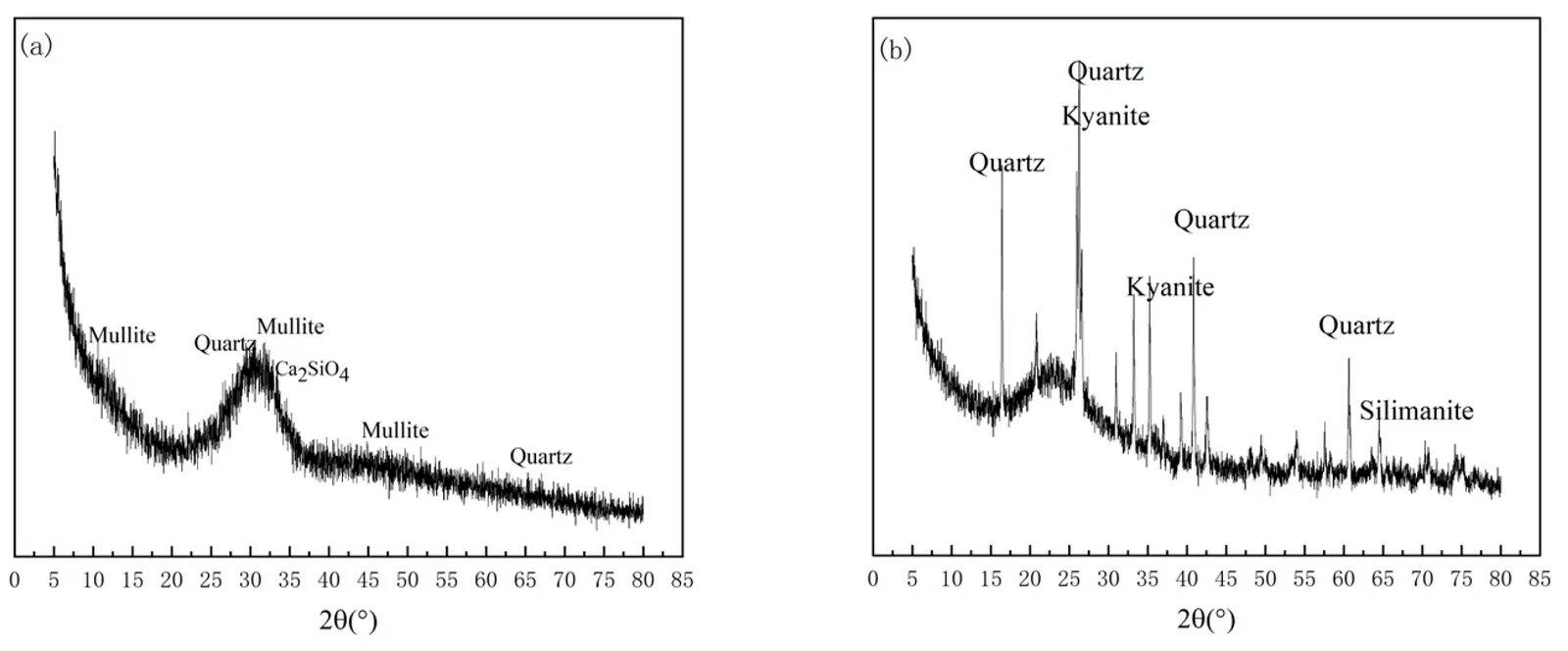
XRD patterns of (**a**) GGBS and (**b**) FA.

GGBS is S95 was purchased from Henan Bangneng Metal Materials Co., Ltd., Zhengzhou, China. FA is Class I, and was obtained from Henan Wuhu Environmental Protection Technology Co., Ltd., Zhengzhou, China. The physical properties of GGBS and FA are shown in Table 1 and Table 2, respectively. The SEM and XRD patterns of GGBS and FA are shown in Figure 1 and Figure 2, respectively.

Natural aggregate was obtained from a sand and gravel quarry in Taizhou with a particle size range of 5–20 mm. Its physical properties are listed in Table 3.

**Figure 3:**
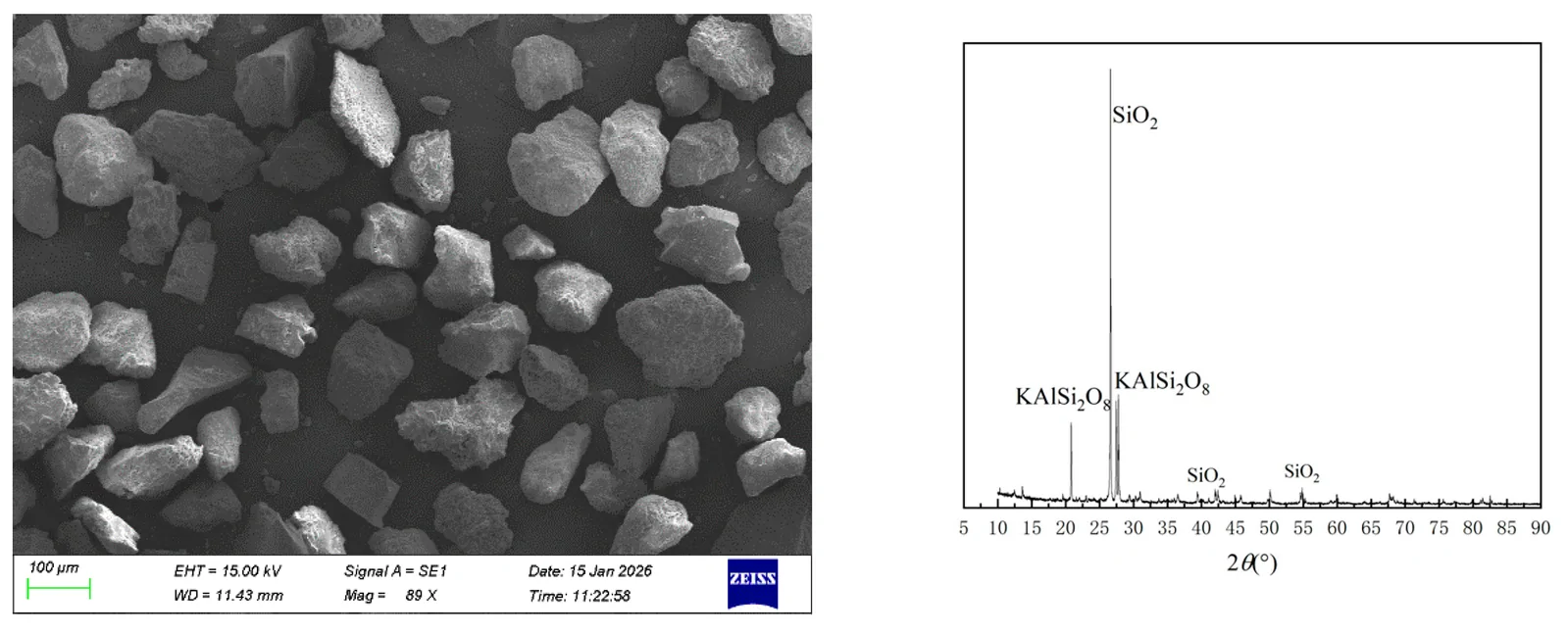
SEM images and XRD patterns of dredged sand.

**Figure 4:**
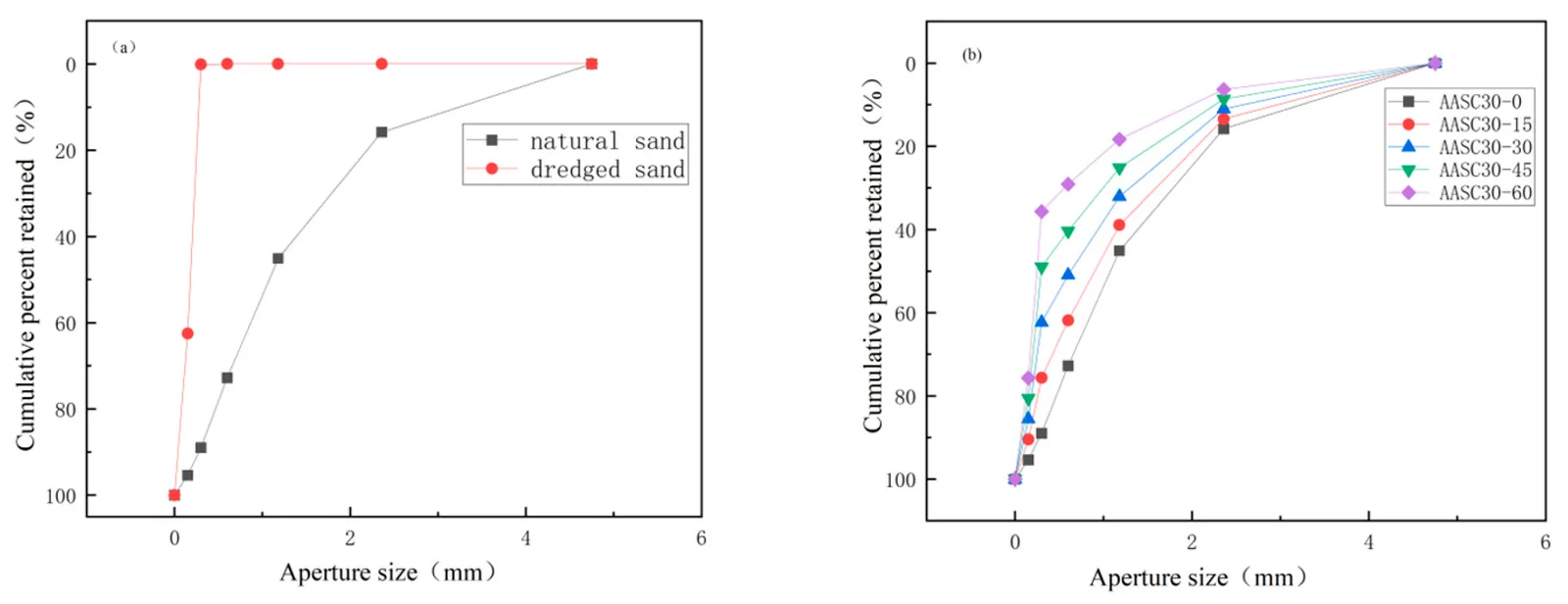
Gradation curve of (**a**) natural sand and dredged sand (**b**) used in the tests.

Fine aggregates include natural sand and Yangtze River dredged sand. Their properties are presented in Table 4.The fineness modulus of natural sand is 3.19. The Yangtze River dredged sand was sampled from the Gaogang area, with a fineness modulus of 0.62, classified as ultrafine sand. Its SEM images and XRD patterns are shown in Figure 3. The particle-size distribution curves of the fine aggregate, dredged sand, and test sand are presented in Figure 4. Compared with ordinary sand, dredged sand has smaller particle sizes and more angular grains, and contains a large number of flaky particles. This particle morphology results in greater interparticle friction.

The alkaline activator is water glass purchased from Jiashan Yourui Refractory Materials Co., Ltd., Jiaxing, China, and its detailed parameters are shown in Table 5. The modulus of sodium silicate was adjusted by adding NaOH. A naphthalene-based superplasticizer at a dosage of 4% and a 2% retarder were incorporated to tailor the setting behavior.

### 2.2. Specimen Preparation and Mix Proportion Design

The mix proportion design of the test referred to Reference [19]. The raw materials adopted were GGBS and FA. The alkali equivalent (Na_2_O) was 5%, the modulus of the activator was 1.0, and the sand ratio was 40%. To investigate the effect of the dredged sand replacement ratio on alkali-activated concrete, five groups with different dredged sand replacement ratios (0%, 15%, 30%, 45%, 60%) were designed. In addition, considering the influence of different FA dosages on the performance of dredged sand concrete, three groups with varying FA replacement ratios (15%, 30%, 45%) were prepared. The detailed mix proportions are listed in Table 6.

### 2.3. Methods

#### 2.3.1. Workability of AASC

Slump was measured in accordance with the requirements of the Standard for Test Method of Performance on Ordinary Fresh Concrete (GB/T 50080-2016) [20].

#### 2.3.2. Mechanical Properties of AASC

The compressive strength and splitting tensile strength of concrete were tested according to the Standard for Test Methods of Physical and Mechanical Properties of Concrete (GB/T 50081-2019) [21]. The compressive strength was tested at 3 d, 7 d, 28 d and 90 d, while the splitting tensile strength was tested at 3 d, 7 d and 28 d. Cubic specimens of 100 × 100 × 100 mm were adopted in the test, with 3 specimens in each group.

#### 2.3.3. Microscopic Properties of AASC

After the mechanical property tests, samples were taken from specimens of corresponding ages. The samples were immersed in anhydrous ethanol to terminate hydration for subsequent microscopic tests. The test methods mainly included XRD, SEM/EDS and MIP. A Zeiss EVO15 scanning electron microscope and Oxford energy-dispersive spectrometer were used to observe the micromorphology of hydration products and the microstructure of the interfacial transition zone. A D8 Advance diffractometer was adopted to analyze hydration products. Mercury intrusion porosimetry (MIP) was performed using a Micromeritics AutoPore IV porosimeter, supplied by Micromeritics Instrument Corporation, Norcross, GA, USA.

## 3. Results and Discussion

### 3.1. Workability

**Figure 5:**
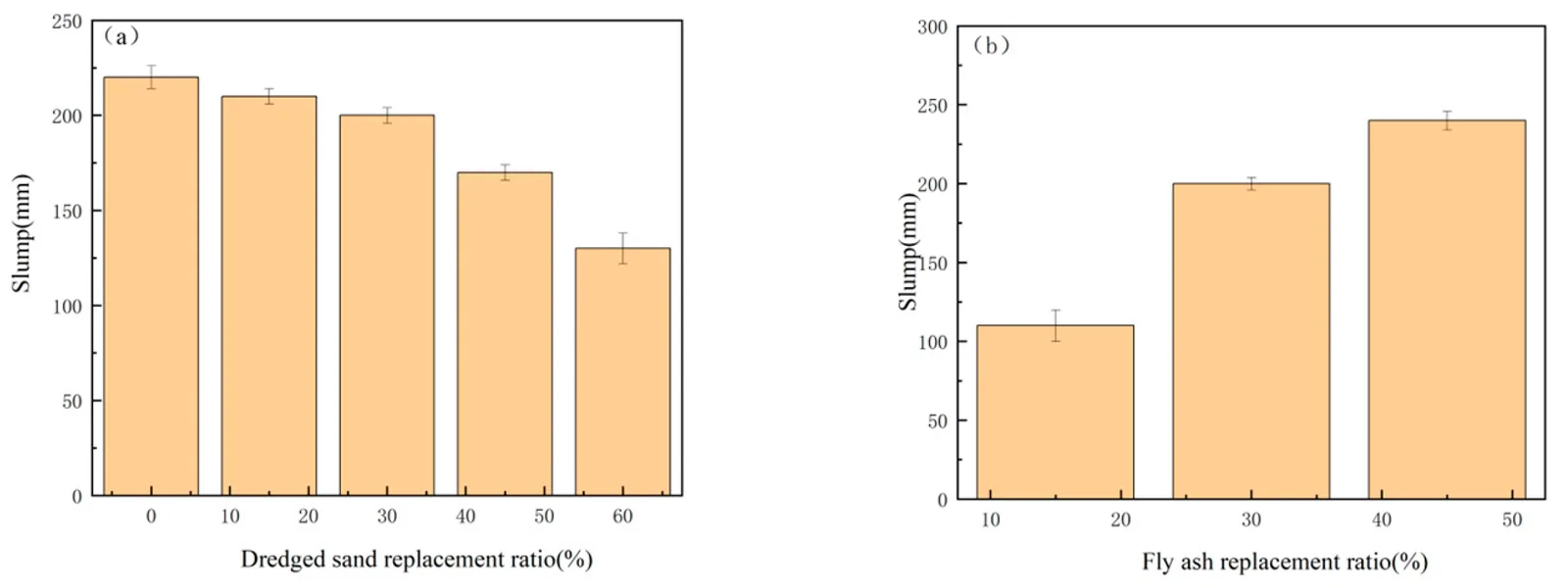
Effects of different factors on the slump of AASC: (**a**) dredged sand and (**b**) fly ash.

Figure 5a shows that the slump of concrete decreases continuously with the increase in the content of dredged sand. This indicates that the incorporation of dredged sand leads to a reduction in concrete workability. A slump value of 220 mm was obtained for the mixture without dredged sand. At a dredged sand content of 15%, the slump decreased to 210 mm. Further increasing the dredged sand replacement led to a marked deterioration in concrete workability, and the slump fell to merely 130 mm at a replacement ratio of 60%. With the mass of cementitious materials kept constant, dredged sand had a low fineness and a large specific surface area, which reduced the content of free water and the effective water–cement ratio, thereby decreasing the slump [22]. Furthermore, Figure 1 reveals that the dredged sand is dominated by irregular particles. The friction between particles and aggregates is therefore increased and leads to the reduction in slump.

It is found that the slump of concrete is positively correlated with the content of FA by varying the FA dosage in the precursor. The slump reaches 110 mm at an FA content of 15%, and 240 mm when the FA content increases to 45%. The SEM images of GGBS show that most GGBS particles are irregular, which increases the friction between particles and aggregates [23]. In addition, GGBS powder possesses high reactivity; under highly alkaline conditions, its dissolution rate is accelerated. When the dissolved Ca^2+^ reacts rapidly with the reactive SiO_2_ in sodium silicate, the aluminosilicate materials dissolve from the raw materials and form a large amount of C-A-S-H gel. This gel coats the particles, thereby significantly reducing the fluidity of the matrix [24,25]. The SEM images of FA show that FA particles are mainly spherical. When incorporated into concrete, FA produces a ball-bearing effect [26], which increases the fluidity of concrete.

### 3.2. Mechanical Properties

#### 3.2.1. Cubic Compressive Strength

**Figure 6:**
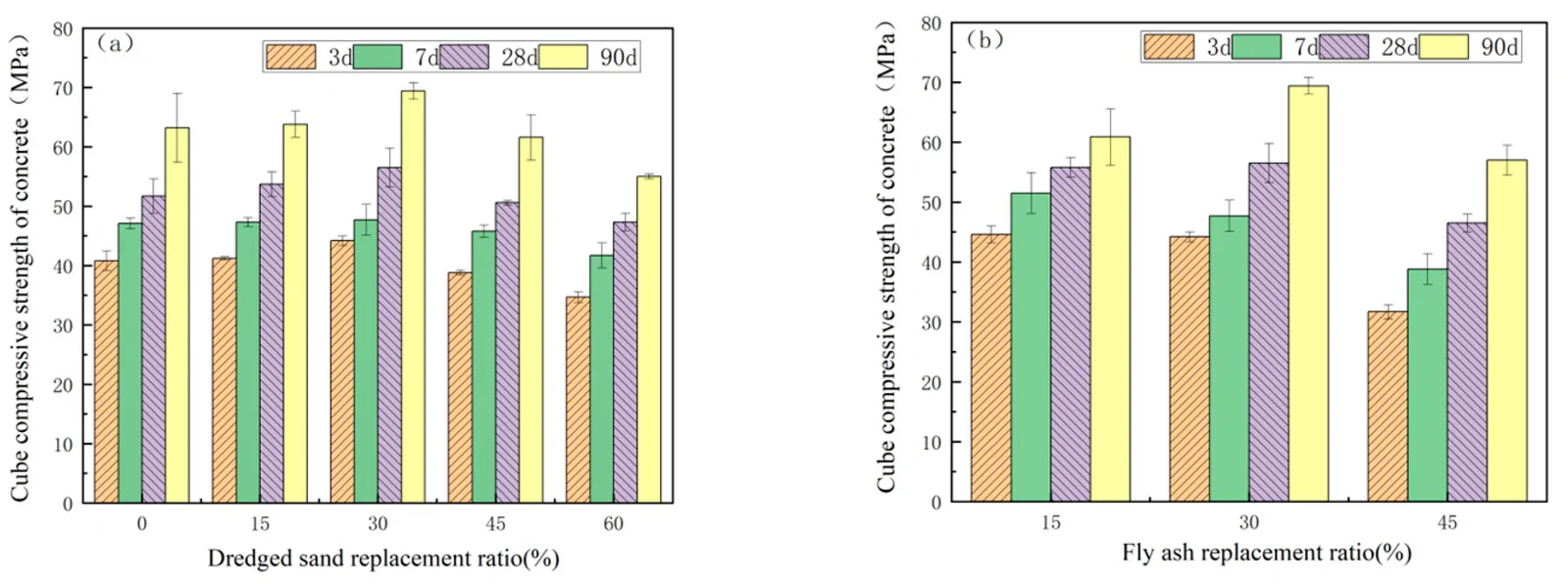
Effects of different factors on the compressive strength of AASC: (**a**) dredged sand and (**b**) fly ash.

Figure 6a shows that the concrete exhibited a relatively high cubic compressive strength at 3 days. It can be seen that the compressive strength of the specimens incorporating dredged sand shows a trend of increasing first and then decreasing. The highest compressive strength of 44.2 MPa is achieved at a dredged sand replacement ratio of 30%, exceeding that of the control mixture without dredged sand by 8.3%. This demonstrates that proper incorporation of dredged sand can enhance the early cubic compressive strength of concrete. This phenomenon can be attributed to the fine particle size and high water absorption of dredged sand. When dredged sand is incorporated into the concrete matrix, it absorbs a large amount of water [27], which reduces the actual water–cement ratio of concrete and leads to a more sufficient hydration reaction in the matrix.

From the cubic compressive strength results at 7 d, 28 d and 90 d, similar effects of dredged sand on the cubic compressive strength of concrete are observed, with the strength exhibiting a trend of first increasing and then decreasing. Among them, the maximum compressive strength is obtained at a dredged sand content of 30%. Compared with the concrete without dredged sand, the compressive strengths at 7 d, 28 d and 90 d are increased by 1.3%, 9.3% and 9.8%, respectively. This can be ascribed to the filling effect of fine particles. The internal porosity of concrete is reduced, and higher mechanical strength is consequently obtained. However, with the increase in dredged sand dosage, the water absorption of aggregate is elevated, leading to an insufficient mortar volume and reduced fluidity of concrete. In this case, concrete cannot be fully compacted during vibration, thereby generating considerable internal pores. For this reason, the compressive strength declines with the further increase in dredged sand content [28].

**Figure 7:**
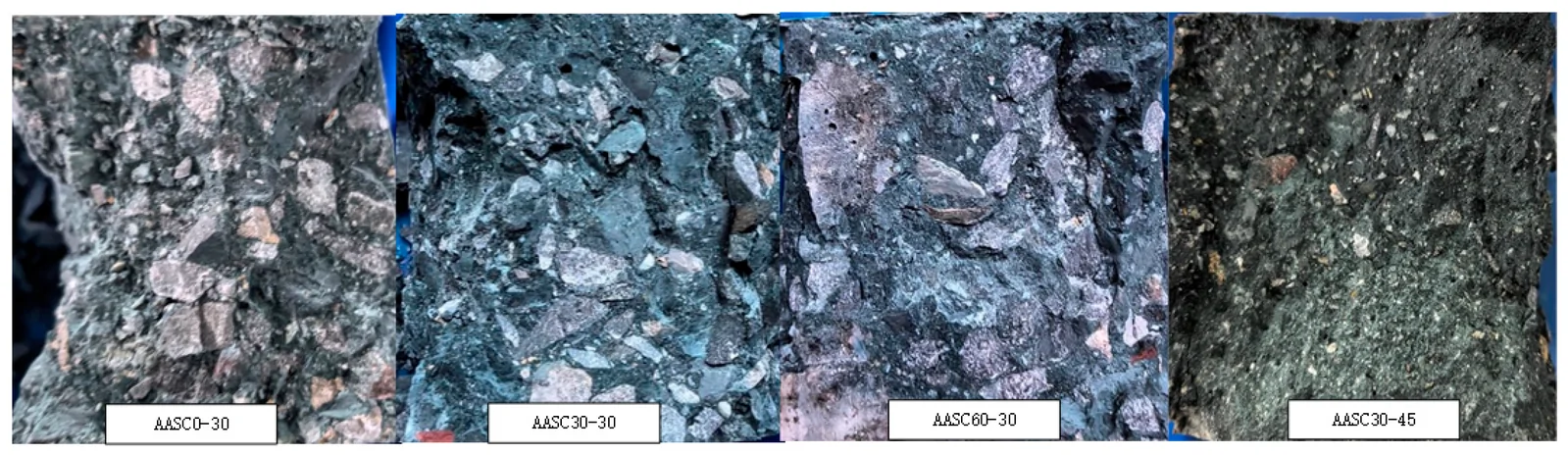
Fracture surfaces of cubic specimens after compressive strength test.

From the fracture micrographs (Figure 7), it is observed that the fracture surface of specimens without dredged sand is relatively smooth, and most aggregates on the surface exhibit fractured failure. As the dredged sand content increases, the matrix fracture surface becomes uneven. Some aggregates are pulled out, and the paste cannot fully wrap the aggregates, resulting in weak interfacial bonding between aggregates and paste. Consequently, the gripping force of hardened paste on aggregates is reduced. Therefore, as the replacement level of dredged sand increases, the number of binding phases produced by hydration reactions between coarse aggregate particles increases accordingly. Such binding phases can fill pores and thereby improve the strength of concrete. Nevertheless, excessive content of these binding phases leads to strength degradation, because the binding matrix formed by dredged sand exhibits a markedly lower strength than the coarse aggregate, and an excessive proportion of this matrix induces interfacial deterioration.

As illustrated in Figure 6b, when the fly ash dosage increases from 15% to 45%, the 3-day and 7-day compressive strengths of concrete show a gradual decreasing trend. This is attributed to the high reactivity of ground granulated blast-furnace slag (GGBS). At the early reaction stage, GGBS is the primary reactive component that generates hydraulic products such as C-S-H and C-A-S-H gels. Consequently, a higher proportion of GGBS contributes to superior early-age strength. The 28-day cubic compressive strength of concrete presents a trend of first increasing and then decreasing as the fly ash content increases from 15% to 45%. Compared with the control group AASC30-30, the cubic compressive strength decreases by 1.2% at a fly ash content of 15%, and a strength reduction of 17.6% is observed when the fly ash content reaches 45%. This indicates that an appropriate incorporation of fly ash is beneficial to the development of cubic compressive strength, whereas adverse effects are induced by excessive fly ash content. This is because fly ash possesses a lower calcium content and exhibits a slower dissolution rate under alkali activation conditions compared with mineral powder. Nevertheless, when an appropriate amount of fly ash is reacted with mineral powder in the matrix, abundant gels are generated in the solution, which can promote the further dissolution of fly ash and thereby improve the mechanical strength [29]. However, as the fly ash dosage is further increased, the alkali activation reaction cannot be fully completed due to the low reactivity of fly ash, which results in a reduction in matrix strength.

**Figure 8:**
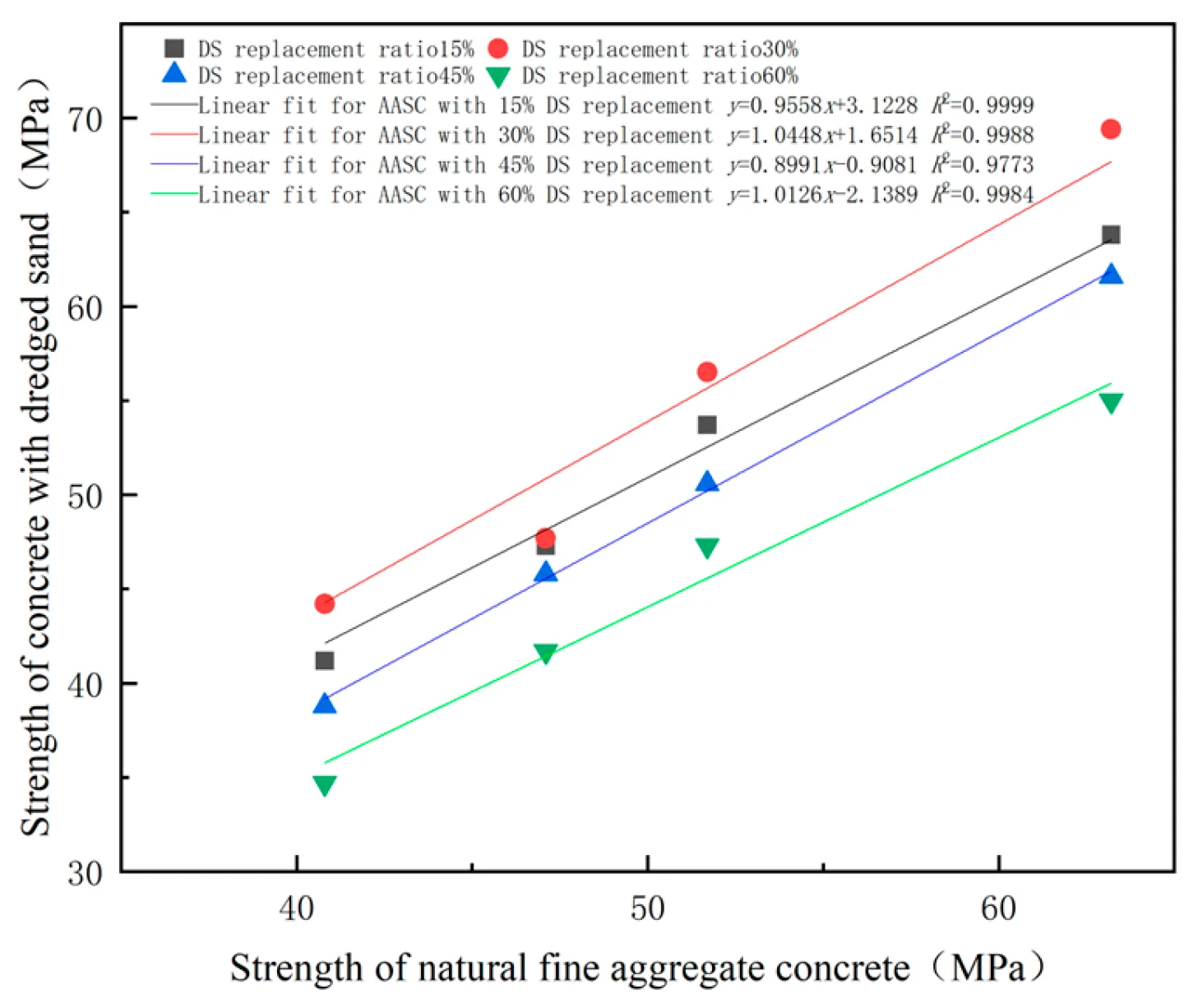
Fitting curves under different replacement rates of dredged sand.

Linear fitting is performed between the cubic compressive strength of AASC with different dredged sand contents and that of concrete using natural fine aggregate. The influence law of different dredged sand replacement ratios on the 3 d, 7 d, 28 d and 90 d cubic compressive strength of alkali-activated concrete can be intuitively analyzed. Fitting parameters for mixtures with different replacement ratios of dredged sand and fly ash are presented in Figure 8, and the fitting equation is shown in Equation (1).

*y* = *ax* + *b* (1)

where *x* is the cubic compressive strength of concrete with natural fine aggregate, *y* is the compressive strength of dredged sand-incorporated AASC, and *a* and *b* correspond to the linear coefficient and constant term of the linear fitting function, respectively. Their values are associated with the dredged sand replacement ratio and curing age.

As shown in Figure 8, good correlations are observed for all curves, with the correlation coefficient ranging from 0.9773 to 0.9994. As the dredged sand content increases from 15% to 60%, a trend of first increasing and then decreasing is observed for the cubic compressive strength of alkali-activated concrete. At dredged sand replacement ratios of 15% and 30%, the intercepts remain above zero, implying that dredged sand contributes positively to the development of cubic compressive strength under these two conditions. This phenomenon may result from the potential pozzolanic property of dredged sand. For dredged sand replacement ratios higher than 30%, the intercept turns negative and decreases gradually with an increasing replacement ratio, indicating that high dosages of dredged sand impose adverse impacts on the cubic compressive strength of concrete. As the dredged sand content increases, the workability of concrete deteriorates, resulting in reduced uniformity and compactness and consequently a decline in compressive strength.

#### 3.2.2. Splitting Tensile Strength

**Figure 9:**
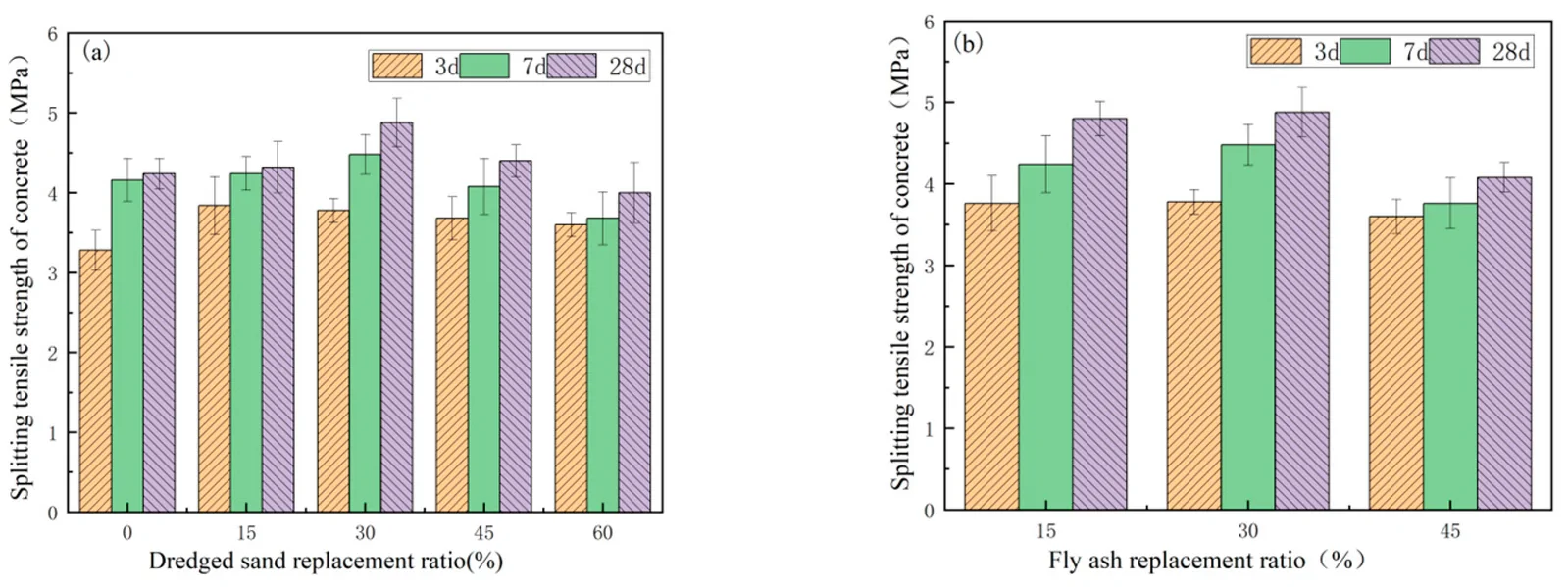
Influence of different factors on splitting tensile strength of AASC: (**a**) dredged sand and (**b**) fly ash.

**Figure 10:**
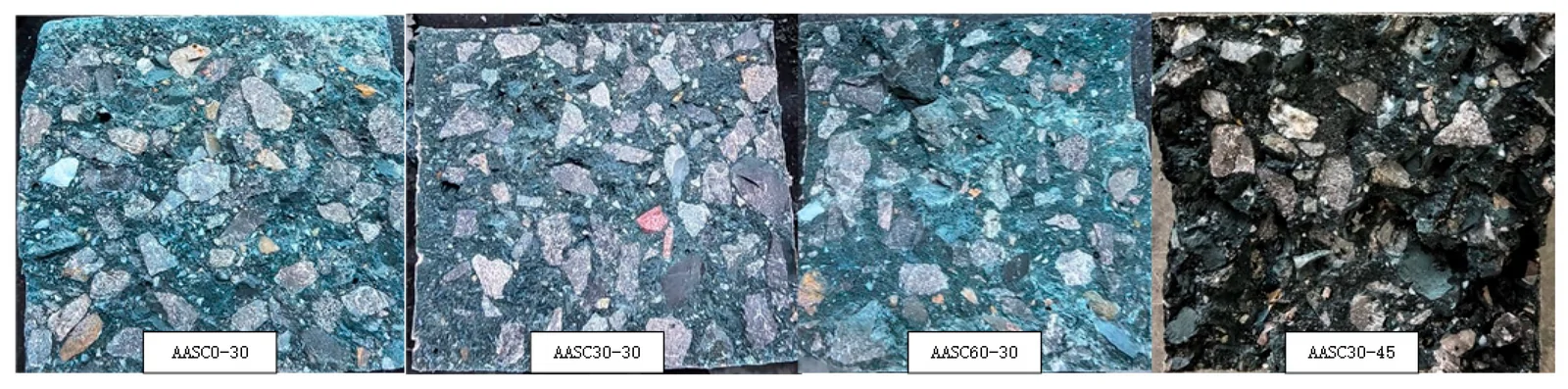
Fracture surfaces after splitting tensile test.

During the splitting tensile test, no notable variation is observed in the specimens before the ultimate load is attained. Sudden failure occurs once the ultimate load is reached; the splitting tensile strength is shown in Figure 9, and the failure surfaces are shown in Figure 10.

As shown in Figure 9a, the splitting tensile strength of concrete exhibits a trend of first increasing and then decreasing with the rise in dredged sand content. For the 3 d splitting tensile strength, the maximum value of 3.84 MPa is obtained at a dredged sand content of 15%, representing a 17% increase compared with the group without dredged sand. For the 28 d splitting tensile strength, the maximum value of 4.88 MPa is achieved at a dredged sand content of 30%, which is 15% higher than that of the reference mixture without dredged sand. As shown in Figure 10, when the dredged sand content is low, the splitting surface is relatively dense. Most coarse aggregates on the failure surface are observed to be directly split, indicating favorable bonding between cementitious materials and aggregates and thus a high splitting tensile strength. It demonstrates that the incorporation of a small amount of dredged sand can fill the internal pores of concrete, making the specimens denser and improving the splitting tensile strength. Nevertheless, with the increase in dredged sand content, the splitting surface appears pulverized. Fracture mainly occurs at the interfacial transition zone between cementitious materials and aggregates. Numerous flaky fragments of hardened cementitious binders and sandy residues are generated after splitting, and the bonding performance is deteriorated. This indicates that excessive dredged sand content exerts a negative effect on the splitting performance. Accordingly, an appropriate dosage of dredged sand can improve the splitting tensile strength of concrete [30].

As shown in Figure 9b, the splitting tensile performance of concrete exhibits a trend of first increasing and then decreasing with varying fly ash replacement ratios. The maximum splitting tensile strength of 4.88 MPa is obtained at a fly ash replacement ratio of 30%. Compared with the group with a 15% fly ash replacement ratio, the increase in the 28-day splitting tensile strength is slight, at only 1.67%. When the fly ash replacement ratio reaches 45%, the splitting tensile strength of concrete is 4.08 MPa, representing a 15% reduction relative to the group with a 15% fly ash replacement ratio.

The ratio of splitting tensile strength to cubic compressive strength of AASC is listed in Table 7. This ratio is generally maintained in the range of 1/11.5–1/12.5, while that of ordinary concrete falls between 1/17 and 1/8 [31]. Compared with ordinary concrete, AASC exhibits relatively low splitting tensile strength, which is consistent with the findings from previous research [27].

### 3.3. Microscopic Properties

#### 3.3.1. Phase Analysis

**Figure 11:**
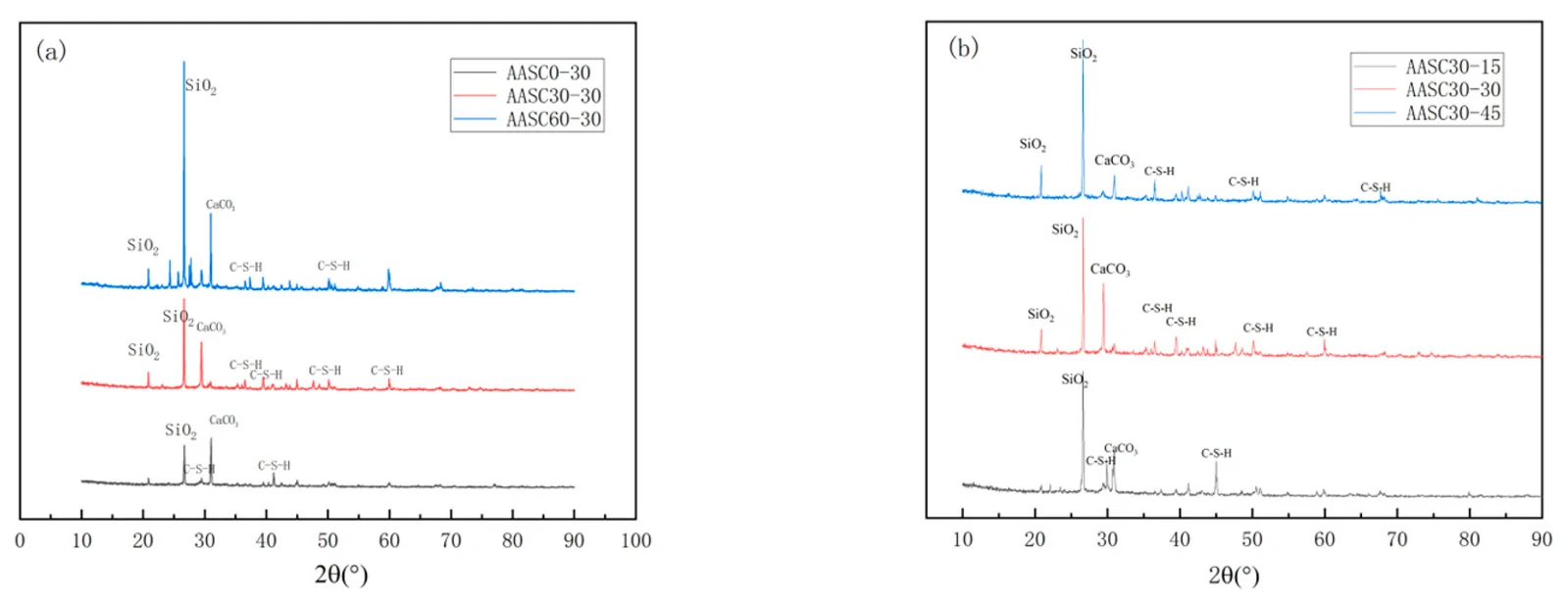
XRD patterns of AASC under different influencing factors: (**a**) dredged sand and (**b**) fly ash.

To further investigate the influences of dredged sand and fly ash dosages on reaction products of hardened specimens, XRD patterns of 28 d cured samples are characterized and presented in Figure 11. The results show that the major diffraction peaks of AASC correspond to SiO_2_, CaCO_3_ and other phases. Among them, the diffraction peak of SiO_2_ is relatively prominent, which corresponds to the sand particles in the mortar. In addition, obvious diffraction peaks of CaCO_3_ are also detected. This phenomenon is attributed to the carbonation of partial C-S-H gel [32,33]. However, it should be noted that no new phases are formed with the variation in precursors and fine aggregates, which is consistent with the findings reported in the literature [34]. It seems that the incorporation of DS and FA does not alter the types of reaction products, though they may affect the efficiency of the hydration reaction and the content of final hydration products. As shown in the figure, the intensity of the CaCO_3_ diffraction peak increases significantly at dredged sand dosages of 30% and 60%. It is suggested that the amounts of C-A-S-H and N-A-S-H phases in concrete are markedly higher than those in the reference group. This is because the potentially active components in dredged sand are more easily activated under alkaline environments, thus accelerating the formation of hydration products. In addition, it is also found that the incorporation of dredged sand may change the concentration distribution of alkaline solution in concrete, further enhancing the alkali activation effect. It can also be observed from Figure 11b that the height of diffraction peaks of hydration products shows a trend of first increasing and then decreasing with the rise in the FA replacement ratio. When the fly ash content reaches 45%, the height of the diffraction peaks of hydration products is lower than that of the group with 30% FA. It is suggested that a 45% FA dosage inhibits the formation of hydration products in AASC, which may account for the reduction in concrete strength.

#### 3.3.2. Morphology and EDS Analysis

**Figure 12:**
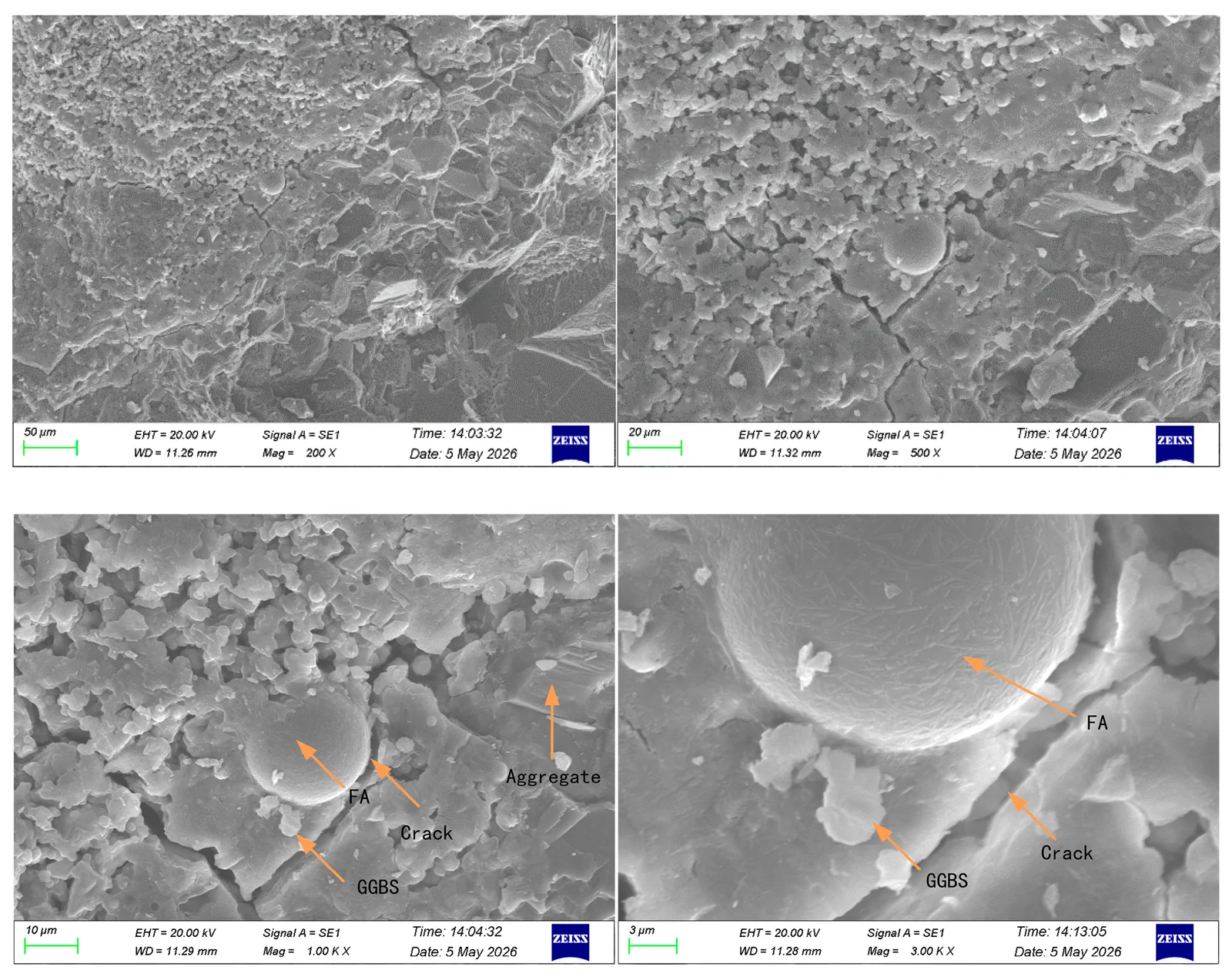
SEM images of AASC-0-30.

**Figure 13:**
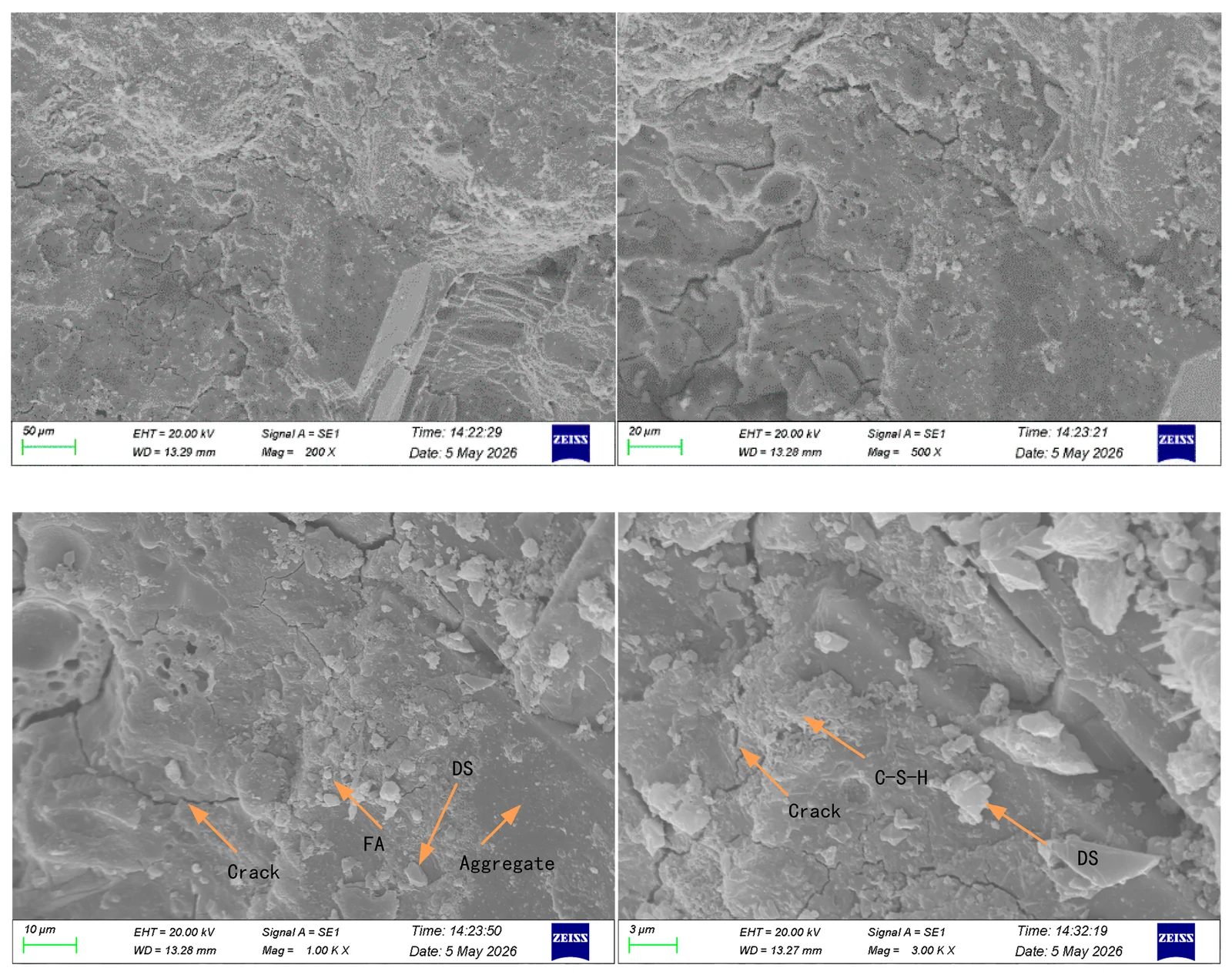
SEM images of specimen AASC-30-30.

**Figure 14:**
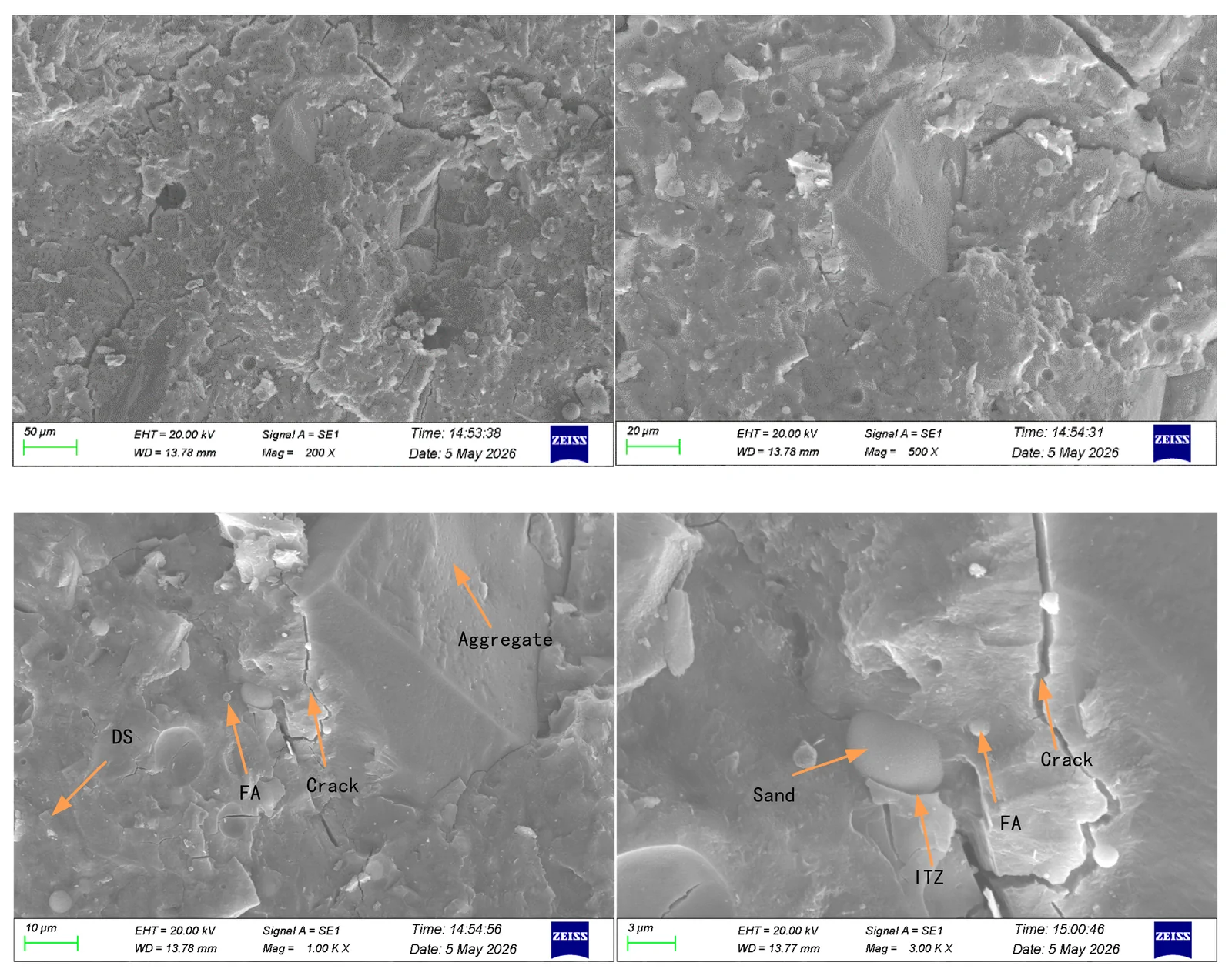
SEM images of specimen AASC-60-30.

**Figure 15:**
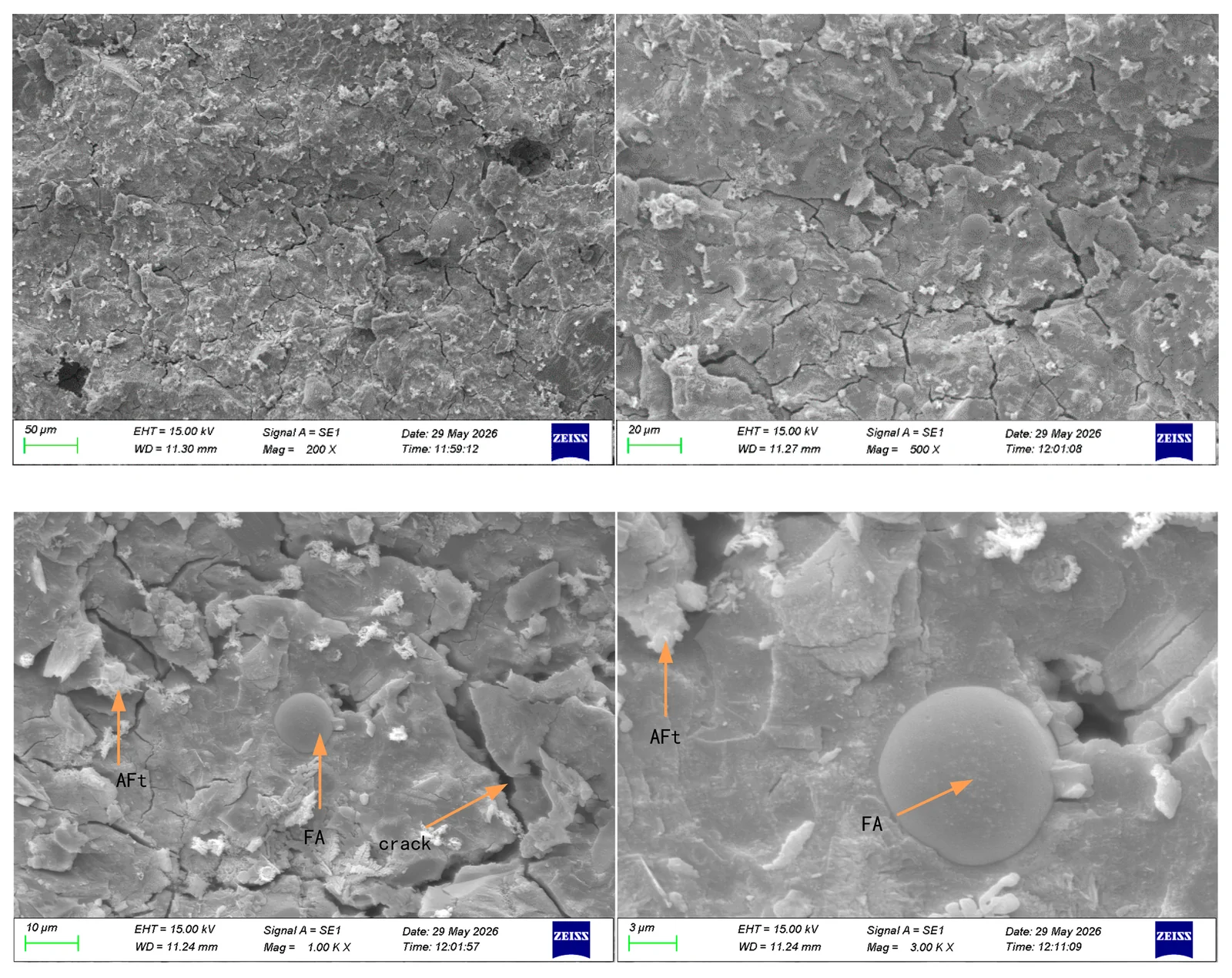
SEM images of specimen AASC-30-15.

**Figure 16:**
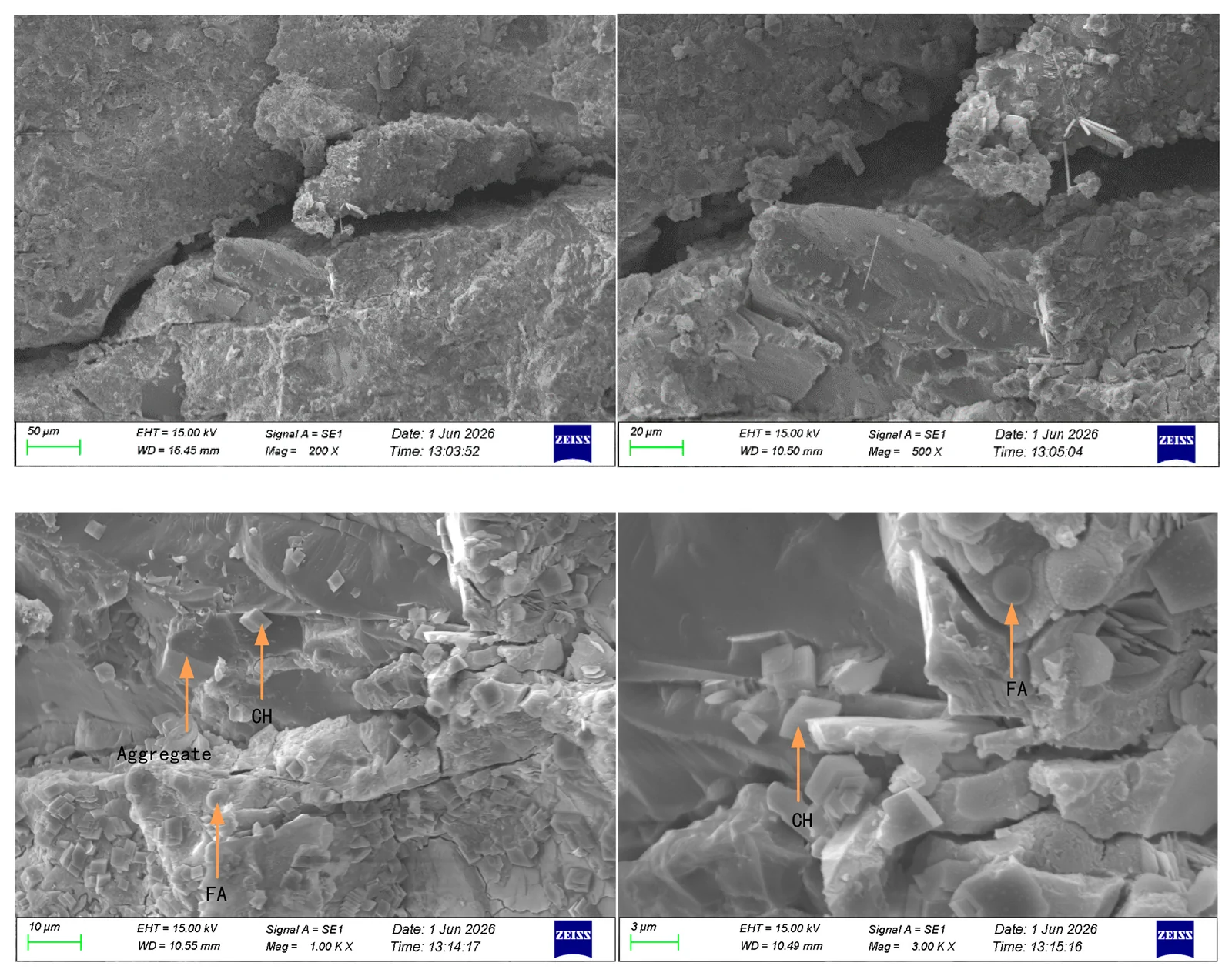
SEM images of specimen AASC-30-45.

To observe the variations in hydration degree and microstructure of AASC under different replacement ratios of dredged sand and fly ash, SEM images of 28 d specimens at various magnifications are shown in Figure 12, Figure 13, Figure 14, Figure 15 and Figure 16, and the EDS results are listed in Table 8.

Figure 12, Figure 13, Figure 14, Figure 15 and Figure 16 display the microstructures of AASC with different dredged sand dosages. Figure 12 corresponds to the reference group without dredged sand, in which some unreacted slag particles can be observed to be wrapped by hydration products. It can be seen from Figure 13 and Figure 14 that when the dredged sand dosages are 30% and 60%, the overall morphology of AASC remains relatively dense. The hydration products uniformly wrap around the aggregates, and the specimens exhibit high compressive strength. However, it can be observed that the microstructure becomes looser with the increase in dredged sand dosage, accompanied by fewer reaction products and thinner gel layers around aggregates. More pores exist in the matrix, which indicates that high dosages of dredged sand exert adverse effects on the microstructure and reduce the strength of AASC.

By comparing Figure 12, Figure 15 and Figure 16 to investigate the microstructure evolution of the matrix with different FA dosages, it can be found that exposed FA particles are more readily observed in the matrix as FA content increases. Nevertheless, unhydrated FA particles are hardly detected at low FA contents. Combined with the mechanical property results, GGBS may promote the dissolution of FA to a certain extent. In the meantime, specimens with low FA content possess a relatively dense matrix with abundant microcracks. It has been reported [35] that this phenomenon can be attributed to the shrinkage generated during GGBS hydration. It can be seen from Table 8 that the Ca/Si ratio of the group with 30% dredged sand is higher than that of the group without dredged sand, demonstrating that appropriate incorporation of dredged sand promotes the formation of Ca/Si gel. Similarly, compared with the group with 15% FA, the specimen group with 30% FA exhibits a higher Ca/Si ratio. It is suggested that proper FA addition facilitates the formation of Ca/Si gel and thereby improves the compressive strength of the matrix.

#### 3.3.3. Pore Structure Analysis

**Figure 17:**
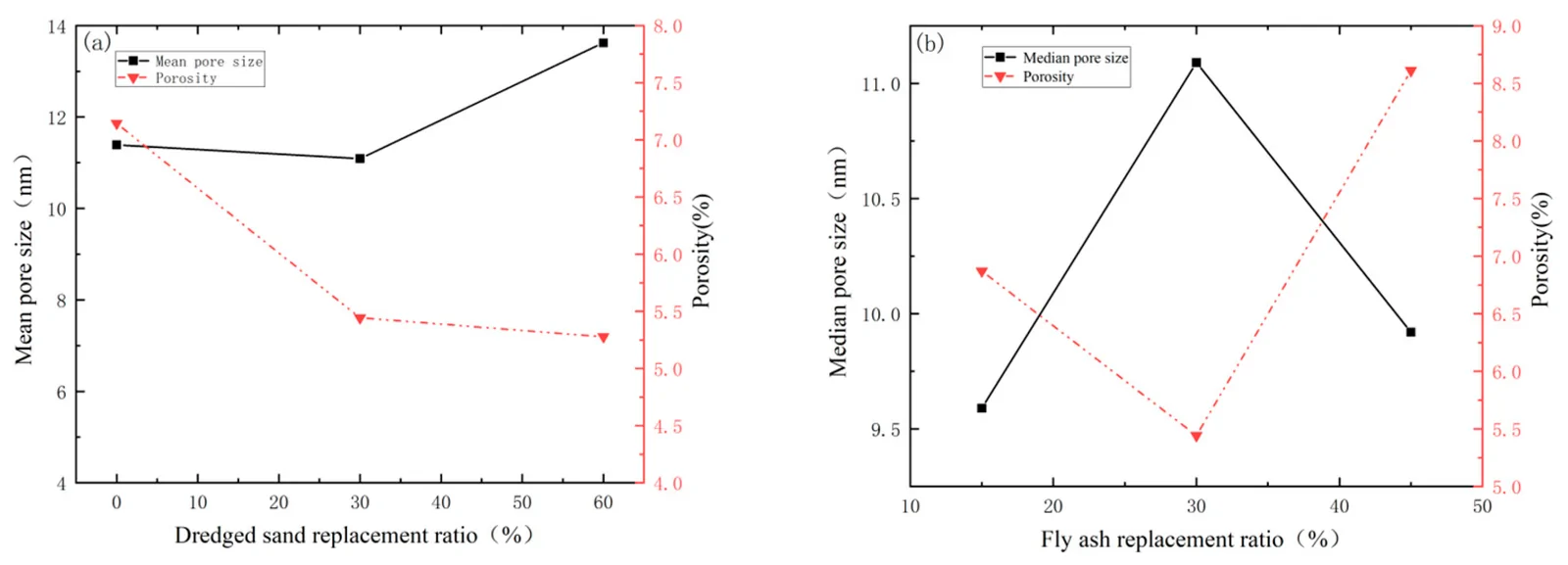
Effects of different factors on average pore size and porosity of AASC: (**a**) dredged sand and (**b**) fly ash.

**Figure 18:**
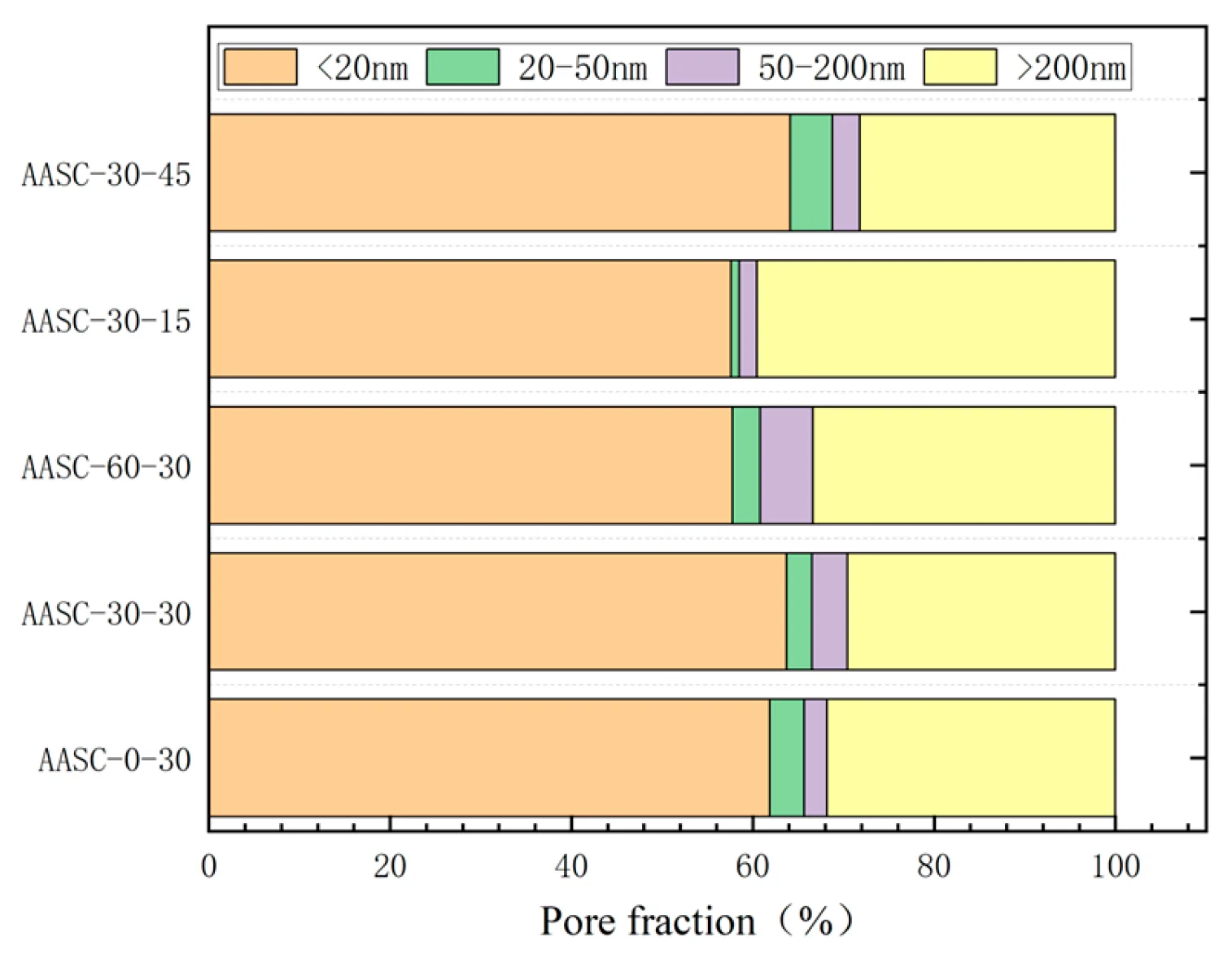
Pore size distribution of AASC under different factors.

**Figure 19:**
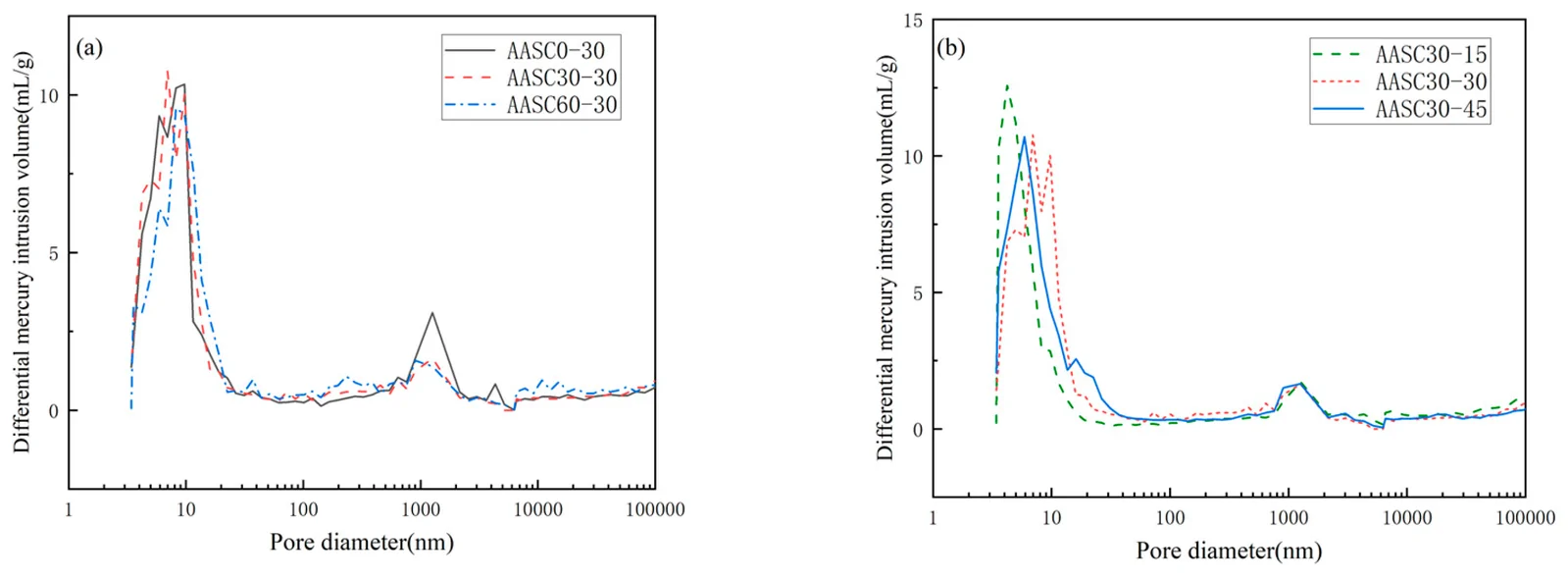
Differential mercury intrusion curves of AASC under different factors: (**a**) dredged sand and (**b**) fly ash.

**Figure 20:**
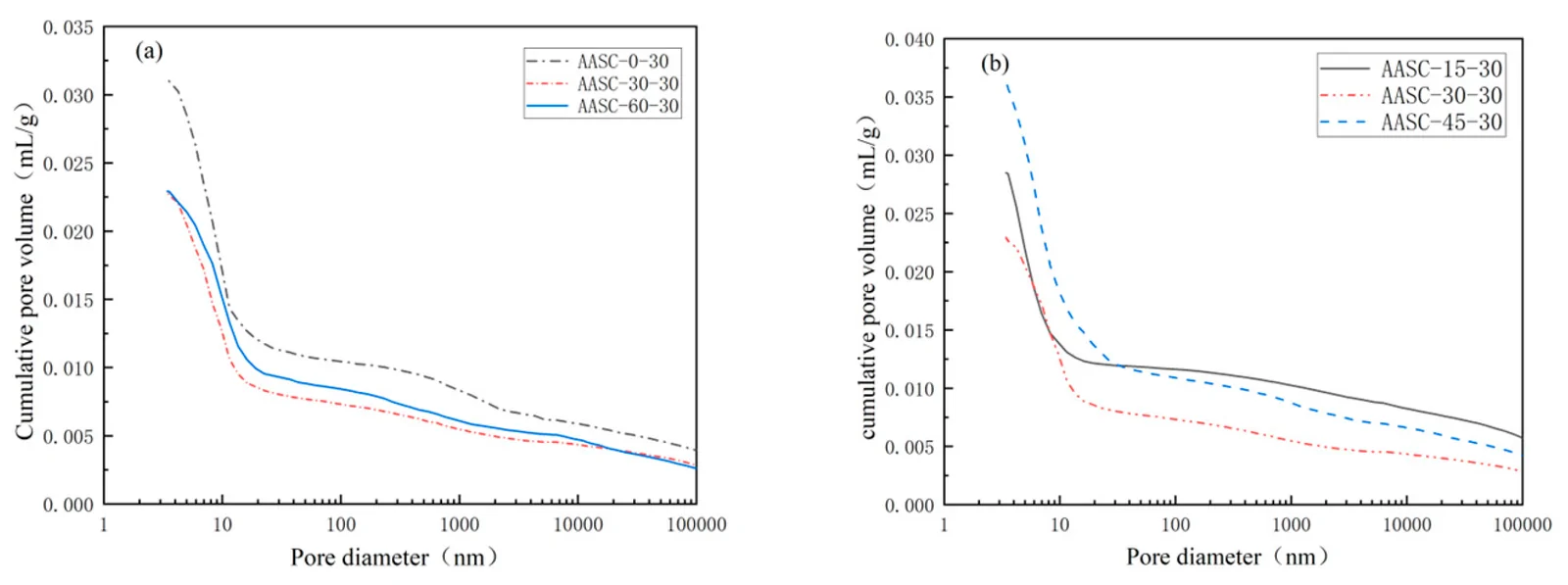
Cumulative pore size distribution curves of AASC under different factors: (**a**) dredged sand and (**b**) fly ash.

The strength of concrete is usually governed by its submicroscopic structure. A reduction in porosity can markedly improve mechanical strength [36]. To investigate the pore distribution of AASC with different mix proportions, mercury intrusion porosimetry (MIP) tests were carried out on 28 d specimens. The average pore size and porosity are presented in Figure 17. Based on the effect of pore size on the properties of AASC, pores are classified into four categories in existing studies: more harmful pores (>200 nm), harmful pores (50–200 nm), less harmful pores (20–50 nm) and harmless pores (<20 nm) [37]. Figure 17 shows the effects of different factors on the average pore size and porosity of AASC. Figure 18 presents the pore distribution of AASC under different factors. Figure 19 illustrates the differential mercury intrusion volume of AASC affected by various factors, and Figure 20 displays the cumulative pore size distribution curves of AASC under the influence of different factors.

As shown in Figure 17a, the porosity of AASC exhibits a decreasing trend as the dredged sand content increases from 0 to 60%, while its average pore diameter decreases first and then increases. When the dredged sand content increases from 0 to 30%, the porosity of AASC decreases from 7.14% to 5.44%, and the average pore diameter drops from 11.39 nm to 11.09 nm. The non-harmful pores increase by 1.8%, the less harmful pores decrease by 1%, the harmful pores rise by 1.4%, and the more harmful pores reduce by 2.2%. As the dredged sand content increases from 30% to 60%, the porosity of AASC decreases from 5.44% to 5.23%, while the average pore diameter rises from 11.09 nm to 13.62 nm. The proportion of non-harmful pores decreases by 5.3%, the fraction of less harmful pores increases by 3%, the proportion of harmful pores increases by 2%, and the fraction of more harmful pores increases by 4.1%. It is suggested that the appropriate incorporation of dredged sand can effectively reduce the porosity and densify the concrete matrix, which may account for the improvement in compressive strength. Nevertheless, excessive addition of dredged sand facilitates the transformation of non-harmful pores into detrimental pores and promotes the formation of large pores, thereby degrading the mechanical strength of concrete.

As shown in Figure 17b, when the FA dosage increases from 15% to 45%, the porosity of AASC exhibits a trend of first decreasing and then increasing, while its average pore size rises first and subsequently declines. When the FA dosage increases from 15% to 30%, the porosity decreases from 6.87% to 5.44%, and the average pore size rises from 9.59 nm to 11.09 nm. The harmless pores increase by 6.1%, less harmful pores increase by 1.9%, harmful pores increase by 2%, and more harmful pores decrease by 10.1%. Compared with the group with a 30% FA dosage, when the FA dosage rises to 45%, the pore distribution is roughly equivalent, whereas the porosity increases to 8.6%. It is suggested that appropriate incorporation of fly ash can reduce the more harmful pores and increase the harmless pores in AASC, making the matrix denser and improving its strength. Nevertheless, a high FA dosage increases the porosity of AASC and hinders strength development. Moreover, the distribution curve in Figure 20b shows that the sample with 45% fly ash has a larger pore volume, which further verifies this conclusion.

## 4. Conclusions

The slump of AASC decreases with the increase in dredged sand dosage and rises as FA content increases. Both the 28 d compressive strength and splitting tensile strength of AASC show a trend of first increasing and then decreasing with rising dredged sand dosage. The incorporation of 30% dredged sand can effectively improve the mechanical properties of AASC. Compared with the reference group, the 28 d and 90 d cubic compressive strengths of AASC increase by 9.3% and 9.8%, respectively. In addition, the incorporation of dredged sand not only accelerates the early-stage strength development of AASC but also facilitates its later strength growth. Considering the workability and strength of concrete comprehensively, the recommended dosage of ultrafine dredged sand is 30%, and the fly ash dosage is 30%.

XRD results suggest that the diffraction peaks of AASC are mainly assigned to phases including SiO_2_ and CaCO_3_. Variations in the dosages of dredged sand and FA do not change the types of hydration products but do affect their amounts. When the dredged sand dosage is 30%, the diffraction peaks of the specimen increase significantly. SEM/EDS results reveal that AASC with appropriate dredged sand incorporation presents a dense microstructure, whereas excessive incorporation may induce more internal pores.

MIP results suggest that the incorporation of dredged sand at an appropriate dosage reduces the matrix porosity, increases the proportion of fine pores, and refines the pore structure of concrete. However, excessive replacement with dredged sand induces the growth of detrimental pores within the concrete matrix. Appropriate incorporation of FA can effectively improve the pore structure of AASC. However, a high FA dosage (45%) leads to an increase in harmful pores.

The conclusions of this study can provide a scientific basis for the application of dredged sand as a replacement for conventional fine aggregates in alkali-activated materials and open up new approaches for the resource utilization of solid wastes. Future studies can further explore the characteristics of dredged sand from different regions, as well as its optimal dosage and mixing ratio in alkali-activated concrete, so as to achieve superior comprehensive performance.

## Figures and Tables

**Table 1:** Parameters of S95 GGBS.

SiO_2_ (%)	CaO (%)	Al_2_O_3_ (%)	MgO (%)	Alkali Content (%)	Glassy Phase Content (%)	Activity Index (7 d) (%)	Activity Index (28 d) (%)
33.06	39.29	15.04	9.96	0.56	99	86	97.5

**Table 2:** Basic properties of FA.

AL_2_O_3_ (%)	SiO_2_ (%)	CaO (%)	Water Requirement Ratio (%)	Loss on Ignition (%)
23	43	5.6	91	3.1

**Table 3:** Physical properties of crushed stone.

Particle Size of Crushed Stone	Apparent Density (kg/m^3^)	Bulk Density (kg/m^3^)	Crushing Index (%)	Needle-Flaky Content (%)
5–20 mm	2662	1530	8.7	2.5

**Table 4:** Properties of fine aggregate.

Fine Aggregate	Apparent Density (kg/m^3^)	Fineness Modulus
dredged sand	2580	0.62
natural sand	2690	3.19

**Table 5:** Parameters of sodium silicate.

Na_2_O (%)	SiO_2_ (%)	Solid Content (%)	Modulus	Baumé Degree (20 °C)
8.53	26.98	35.5	3.3	38.5

**Table 6:** Mix proportions of alkali-activated concrete (kg/m^3^).

NO.	Coarse Aggregate	DS	Natural Sand	FA	GGBS	Sodium Silicate	NaOH	Water	Water Reducer	Retarder
AASC0-30	1086	0	724	120	280	70.9	18	130.2	16	10
AASC15-30	1086	109	615	120	280	70.9	18	130.2	16	10
AASC30-30	1086	217	507	120	280	70.9	18	130.2	16	10
AASC45-30	1086	326	398	120	280	70.9	18	130.2	16	10
AASC60-30	1086	434	290	120	280	70.9	18	130.2	16	10
AASC30-15	1086	217	507	60	340	70.9	18	130.2	16	10
AASC30-45	1086	217	507	180	220	70.9	18	130.2	16	10

Note: Taking AASC0-30 in the table as an example for the specimen numbering rule, “0” indicates that the replacement ratio of dredged sand for natural sand is 0%, and “30” denotes that the fly ash content in dredged sand concrete is 30%.

**Table 7:** Relationship between compressive strength and splitting tensile strength of AASC.

NO.	28-Day Splitting Tensile Strength (Mpa)	28-Day Cubic Compressive Strength (Mpa)	Splitting Tensile Strength/Cubic Compressive Strength
AASC0–30	4.24	51.7	1/12.2
AASC15–30	4.32	53.7	1/12.5
AASC30–30	4.88	56.5	1/11.6
AASC45–30	4.40	50.6	1/11.5
AASC60–30	4.00	47.3	1/11.9
AASC30–15	4.80	55.8	1/11.7
AASC30–45	4.08	46.5	1/11.4

**Table 8:** EDS energy spectrum analysis (mass fraction, %).

No.	C	O	Na	Mg	Al	Si	K	Ca	Fe	Ca/Si
AASC-0-30	14.05	47.9	3.15	3.82	5.42	9.64	0.57	14.78	0.68	1.53
AASC-30-30	11.89	32.98	3.79	3.19	5.57	9.9	0.63	28.57	3.48	2.88
AASC-60-30	9.77	45.34	2.08	1.73	3.92	22.66	0.42	13.49	0.6	0.60
AASC-30-15	15.19	30.8	7.17	2.13	5.97	14.12	1.72	21.72	1.17	1.54
AASC-30-45	14.93	38.3	2.59	0.82	3.73	26.69	0.36	11.89	0.69	0.44

## Data Availability

The original contributions presented in this study are included in the article. Further inquiries can be directed to the corresponding author.
